# Microbiota Composition in Diverticular Disease: Implications for Therapy

**DOI:** 10.3390/ijms232314799

**Published:** 2022-11-26

**Authors:** Antonio Tursi, Valerio Papa, Loris Riccardo Lopetuso, Carlo Romano Settanni, Antonio Gasbarrini, Alfredo Papa

**Affiliations:** 1 Territorial Gastroenterology Service, ASL BAT, 70031 Andria, Italy; 2 Department of Translational Medicine and Surgery, School of Medicine, Catholic University, 00168 Rome, Italy; 3 Digestive Surgery, Fondazione Policlinico Universitario “A. Gemelli”, IRCCS, 00168 Rome, Italy; 4 Center for Diagnosis and Treatment of Digestive Diseases, CEMAD, Gastroenterology Department, Fondazione Policlinico Gemelli, IRCCS, 00168 Rome, Italy; 5 Department of Medicine and Ageing Sciences, “G. d’Annunzio” University of Chieti-Pescara, 66100 Chieti, Italy; 6 Center for Advanced Studies and Technology (CAST), “G. d’Annunzio” University of Chieti-Pescara, 66100 Chieti, Italy

**Keywords:** diverticulosis, diverticular disease, acute diverticulitis, gut microbiota, probiotics

## Abstract

Gut microbiota (GM) composition and its imbalance are crucial in the pathogenesis of several diseases, mainly those affecting the gastrointestinal tract. Colon diverticulosis and its clinical manifestations (diverticular disease, DD) are among the most common digestive disorders in developed countries. In recent literature, the role of GM imbalance in the onset of the different manifestations within the clinical spectrum of DD has been highlighted. This narrative review aims to summarize and critically analyze the current knowledge on GM dysbiosis in diverticulosis and DD by comparing the available data with those found in inflammatory bowel disease (IBD). The rationale for using probiotics to rebalance dysbiosis in DD is also discussed.

## 1. Introduction

Diverticulosis of the colon is an anatomical condition implying herniation of the colonic layers (generally, mucosa and submucosa in the left colon and all mucosal layers in the right colon) through points of weakness in the intestinal wall, with protrusion of the gut lumen [1]. Diverticulosis is mostly an asymptomatic condition but may become symptomatic in about 20% of cases, causing so-called diverticular disease (DD) [1]. Most DD patients have symptomatic uncomplicated diverticular disease (SUDD), characterized by symptoms linked to the diverticula but without visible evidence of acute inflammation. Only a minority will develop acute diverticulitis (AD), characterized by acute inflammation of the diverticula [1]. Although the prevalence of SUDD and AD are lower than expected [2,3,4], we do not yet know why some patients with diverticulosis develop symptoms while others do not. There are data in the recent literature pointing out the possible role of gut microbiota (GM) as a critical factor in the pathogenesis of several gastrointestinal (GI) and extraintestinal (EI) diseases, improving the understanding of the highly complex interaction between GM and innate and adaptive immunity in regulating inflammation [5]. Thus, we know that dysbiosis, defined as reduced GM biodiversity in terms of the reduction/increase in some species/strains or an imbalance between symbionts and pathobionts, may be an active modulator of complex pathophysiological processes [6]. Microbiota imbalance has also been proposed as a mechanism to explain the symptoms of DD [1]. However, knowledge of GM in DD is still currently limited, and its role in the pathogenetic cascade of the disease is under active debate. The present narrative review aims to critically summarize the current state-of-the-art literature on the association between GM, diverticulosis, and DD while also providing some suggestions about the manipulation of GM as a possible therapeutic option in these patients.

For this purpose, a literature search was conducted using PubMed for publications up to September 2022. Original articles and reviews were identified using the keywords “diverticulosis,” “diverticular disease,” “symptomatic uncomplicated diverticular disease,” “acute diverticulitis,” and “complicated diverticulitis,” matched with each of the following keywords: “gut microbiota,” “dysbiosis,” “microbiota imbalance,” and “probiotics.” Additional articles were identified by reviewing the reference lists of selected pertinent articles.

## 2. Profiling the GM in Diverticulosis

Currently, the GM composition in patients with diverticulosis as the primary endpoint has been profiled in only two studies (Table 1). Both studies analyzed mucosa-associated microbiota using PCR metagenomics [7] and 16S rRNA microbial profiling [8]. Although these studies had significantly different sample sizes, neither found a significant microbial imbalance in people with diverticulosis compared with controls [7,8]. However, one exciting feature arose from the study of Jones et al. [7], who found a slight but not significant reduction in *Proteobacteria* and *Comamondaceae* in their patients.

## 3. Profiling the GM in SUDD

The GM in patients with SUDD as the primary endpoint has been profiled in nine studies (Table 2), published from 2016 to 2022. In a small series of four patients with SUDD, compared with other diseases and investigated by stool analysis based on the analysis of 16S rRNA microbial profiling, Ponziani et al. detected an increased abundance of *Roseburia* and *Colinsella* and a decreased abundance of *Lactobacilli* before treatment with rifaximin [9]. Tursi et al. [10] analyzed fecal microbiota from 15 patients with SUDD, 13 subjects with asymptomatic diverticulosis, and 16 healthy people using real-time PCR. The authors identified no differences in the abundance of the main microbiota components among the three groups, except for *A. muciniphila*, which showed an increased abundance in SUDD patients. Significantly, this trend was associated with a different fecal metabolomics profile, including lower levels of N-acetyl compounds and isovalerate in SUDD [10]. Barbara et al. [11] analyzed mucosa-associated colonic microbiota from 8 SUDD patients, 16 subjects with asymptomatic diverticulosis, and 14 controls without diverticulosis. They found no significant differences in the overall fecal microbiota composition between the three groups. Still, subjects with diverticula had a significantly lower abundance of *Clostridium cluster IV* than controls, while patients with SUDD also had a reduced abundance of *Fusobacterium* and *Lactobacillaceae* [11]. Finally, the abundance of *A. muciniphila* was higher in diverticular than non-diverticular colonic districts [11]. Kvasnovsky et al. [12] analyzed fecal microbiota from 28 SUDD (with and without previous acute diverticulitis episodes) using 16S rRNA microbial profiling. *Bacteroides*, *Faecalibacterium*, and *Ruminococcus* were increased in all SUDD patients; a higher bloating severity score was associated with the relative abundance of *Ruminococcus* (positive association) and *Roseburia* (negative association), while the intensity of pain was significantly correlated with the relative abundance of *Cyanobacterium*. *Pseudobutyrivibrio*, *Bifidobacterium*, *Christensenellaceae*, and *Mollicutes F9* increased dramatically in SUDD patients with previous AD episodes versus SUDD patients with no history of AD. Finally, GM biodiversity was positively correlated with fecal calprotectin [12]. Another small study by Lopetuso et al. on stool samples of SUDD patients found that the abundance of *Ruminococcaceae* increased with the depletion of *Colinsella* and *Bacterioidetes fragilis* [13]. Linninge et al. [14] performed an analysis of mucosa-associated colonic microbiota. They enrolled 16 SUDD patients and 35 controls and found that *Enterobacteriaceae* abundance was significantly increased in patients compared with controls. Laghi et al. [15] focused their attention on the impact of treatments on GM. Analyzing stools from 15 patients with SUDD, 13 subjects with asymptomatic diverticulosis, and 16 healthy people using real-time PCR, the authors drew attention to three notable findings: *A. muciniphila* was more abundant in SUDD than in the control group; its abundance decreased during treatments and increased again during the pharmacological washout period, and the abundance of *A. muciniphila* is significantly linked with the severity of abdominal pain [15]. Remarkably, this trend was again associated with a different fecal metabolomics profile [15]. The two more recent studies focused on the abundance of *F. prausnitzii* in these patients. In particular, attention was given to this species because of the earlier finding that its abundance is significantly reduced in inflammatory bowel disease (IBD), playing a possible pathogenetic role in its occurrence [16]. Ponziani et al. [17] treated 25 patients (7 with SUDD) with rifaximin 1200 mg/day for ten months. Forty percent of patients responded to the treatment, with a significant increase in *Faecalibacterium* abundance and a substantial decrease in *Roseburia* and *Ruminococcus* post-treatment. The clinical symptoms improved according to the rise in *Faecalibacterium* abundance [17]. Finally, Tursi et al. [18] analyzed the abundance of *F. prausnitzii* in stored stool samples from patients evaluated in a previous study [10]. The abundance of *F. prausnitzii* was not decreased in the SUDD population but slightly increased.

## 4. Profiling the GM in AD

The GM in patients with AD as the primary endpoint has been profiled in four studies (Table 3), which covered a period from 2007 to 2022. Gueimonde et al. [19] analyzed the mucosa-associated microbiota on a surgical specimen of 9 patients with AD, 21 with colorectal cancer (CRC), and 4 with IBD. Focusing their attention on the different bifidobacterial groups and species in the analyzed mucosal samples, the authors found a significantly higher abundance of *Bifidobacterium longum* in AD than in the other two patient groups (*p* < 0.05) [19]. Daniels et al. [20] analyzed the DNA samples of 31 acute uncomplicated diverticulitis (AUD) patients and 25 controls taken by rectal swab. Using a PCR-based technique and a cross-validated partial least squares discriminant analysis (PLS-DA), the authors found that the ratio of *Firmicutes*/*Bacteroidetes* and abundance of *Proteobacteria* were comparable among patients and controls (*p* = 0.20). Higher diversity in AD for *Proteobacteria* (*p* < 0.00002) was also found, and this discriminative ability was found to be mainly due to species of the family *Enterobacteriaceae* [20]. Schieffer et al. [21] analyzed the differences in the mucosa-associated microbiota of the colon between AD-affected tissue and the adjacent tissue not involved in the inflammatory process. They found that *Microbacteriaceae* were more abundant in the affected mucosa. Moreover, they also analyzed the fungal species in these districts, finding that *Ascomycota* was enriched in the inflamed mucosa. In contrast, three *Basidiomycota species* (*Plutaceae*, *Pluteus*, and *Agaricales*) were more abundant in the adjacent tissue [21]. A more recent study was performed by O’Grady et al. [22]. They conducted a case-control study, enrolling 55 AD patients (44 with AUD and 11 with acute complicated diverticulitis, ACD) compared with 27 controls. In these populations, a rectal swab was performed for 16S rRNA analysis. The *Actinobacteria* and *Proteobacteria* phyla were more abundant in AD than in controls. Representation of the genera *Lachnospiraceae*, *Ruminococcus,* and *Faecalibacterium* decreased in AD, while *Fusobacteria*, *Prevotella*, and *Paraprevotella* representation increased [22]. Interestingly, significant differences were also found between AUD and ACD; in particular, the authors found that *Prevotella*, *Fusicatenibacter*, and *Faecalibacterium* were more abundant in ACD than in AUD [22].

## 5. Rationalization of the GM Changes in DD Compared with Chronic Intestinal Inflammation

As previously reported, there are increasing studies on GM composition in DD. They have tried to identify specific microbiota changes linked to the occurrence of symptoms and disease severity. Although limited by several biases (the low sample size of SUDD patients and healthy control groups, heterogeneity in the sampling methods, and methods for bacteria detection), the results suggest that specific fecal microbiome changes mirror the severity of symptoms and the level of inflammation. The first exciting finding was that asymptomatic diverticulosis is not linked to significant GM alterations. As reported in Table 1, two studies analyzing the mucosa-associated microbiota failed to find any significant alteration in these patients, except for a slight but insignificant reduction in *Proteobacteria* and *Comamondaceae* [7,8]. These findings probably mean that GM changes do not play any role in diverticulosis, in which genetics and the modifications of the characteristic of the colonic wall are still the most important factors [1]. More critical data have arisen from the analysis of SUDD microbiota. As shown in Table 2, the main finding is that not only are some anti-inflammatory and immuno-regulatory taxa such as *Clostridium cluster IV* and *Lactobacillaceae* [23,24,25] decreased, but also that some species with the same activity, such as *A. muciniphila* and *Roseburia*, are increased [1,10,15,17,18]. These dysbiotic landmarks seem to differ from those typically seen during chronic intestinal inflammation, such as in IBD, where a reduced abundance of *Akkermansia*, *Roseburia*, and *Faecalibacterium* is strongly associated with disease severity [26]. In this scenario, *A. muciniphila* is critical in maintaining intestinal health and host metabolic modulation. *A. muciniphila* is a symbiotic member of the gut microbiota belonging to the Verrucomicrobia phylum and is found to be correlated with several pathological conditions [27]. This bacterium is essential in maintaining intestinal homeostasis thanks to its strong interplay with the host cells and the gut microbial community. It is crucial in guaranteeing proper mucus production and thickness [28,29]. The ability to degrade the mucus also has other beneficial effects since it produces oligosaccharides, amino acids, propionate, acetate, and essential vitamins and cofactors, which become useful for other microbial commensals [30,31].

Interestingly, *A. muciniphila* is correlated with several disorders that share a systemic inflammatory background and gut barrier impairment [28,32,33]. *A. muciniphila* decline may represent a definitive biomarker of dysbiosis shared by patients with different GI and EI diseases. It may be the most relevant discriminating factor to dissect the complex equilibrium between health and disease status. This feature may thus have potential diagnostic and therapeutic consequences [34].

Moreover, the kinetics of some species are difficult to explain. For example, *Ruminococcus gnavus*, abundant in IBD and associated with disease activity [35], seems to be decreased in SUDD [17], but its relative abundance seems strongly inversely related to the bloating score [12]. Additionally, the results arising from GM in AD are conflicting. The studies analyzing stool and mucosa-associated microbiota found a relative abundance of taxa exhibiting anti-inflammatory properties (such as *Bifidobacteria*) [19] and pro-inflammatory activity (such as *Enterobacteriaceae*) [20]. Furthermore, even when a more recent analysis found a significantly reduced abundance of *Faecalibacterium* in AD [22], a sub-analysis found that this genus is more abundant in ACD than in AUD [22]. However, the significance of these findings is still unclear, and the definitive role of microbiota changes in those patients should be clarified in future studies. The GM composition observed in DD patients and the reported differences from IBD should be analyzed considering at least four variables, including the type of inflammatory pathway, age of patients, diet, and therapeutic armamentarium. These different variables are discussed below, and some assumptions are given.

### 5.1. Inflammation

The first hypothesis to explain the different characteristics of the intestinal microbiota among patients with DD and IBD concerns the pathogenetic mechanisms determining inflammation. We generally reasoned that DD and IBD, affecting the same organ (the colon), should track the same inflammatory pathway. This consideration arose from the discovery that in DD, as well as IBD, there is overexpression of the tumor necrosis factor (TNF)-α [36,37] and a pro-fibrotic pattern that resembles that found in Crohn’s disease (CD) [38]. However, the inflammatory pathway cannot be the same. For example, the genetic haplotypes of TNF-α [39] and prostaglandin E_2_ expression [40] differ between DD and IBD.

Moreover, interleukin-10 (IL-10), a potent anti-inflammatory regulatory cytokine, is significantly decreased in active IBD [41], does not decrease in SUDD [42], and is moreover increased in post-diverticulitis SUDD [43]. These findings show that there cannot be a complete overlap of the inflammatory mechanisms involved in DD and IBD’s pathogenesis. Consequently, the microbiota composition may differ.

### 5.2. Age

The activity and composition of the human microbiota are known to be influenced by several factors (genetic background, age, diet, and host health status). Moreover, its composition is generally stable, but some changes are evident throughout life and may influence host metabolism and disease development. For example, aging is associated with reduced biodiversity of the GM and increased inter-individual variability [44]. Moreover, microbiota resilience to stressors, such as antibiotic treatments or nonsteroidal anti-inflammatory drugs (NSAIDs), is also reduced, leading to the depletion of taxa with anti-inflammatory activities and overgrowth of pathobionts [44,45]. For example, the abundance of *Firmicutes taxa* significantly increases through life, being higher in adults and the elderly, while *Bifidobacteria* are more abundant at younger ages [46]. Finally, Biagi et al. analyzed these changes in greater depth [47]. They found that *A. muciniphila* is significantly increased in elderly and older adults, with a relationship between its abundance and a chronic inflammatory state [47]. Moreover, since the association between aging and diverticulosis is well known, and the incidence peaks in older ages [1], the findings reported in these studies may explain the relative abundance of this species in DD.

On the other hand, IBD patients are generally younger than DD patients, which could be a further factor that partly explains the difference in GM composition between these two diseases.

### 5.3. Diet

For many years, the risk of developing diverticulosis has been associated with low fiber intake and excessive red meat consumption. However, it is well known that these dietary habits can influence microbiota expression. In animal models, low-fiber diets are more often related to the abundance of *Enterobacteriaceae* and *Bacteroides* than high-fiber diets [48]. In contrast, the habitual human consumption of a low-fiber diet leads to a depletion of *Clostridia*, *Actinobacteria* [49], and all butyrate-producing taxa [50]. High-protein diets generally determine the abundance of bacteria with protein-fermenting capacities, including many *Enterobacteriaceae*, *Streptococcus*, and *Bacteroides*, and the reduction of saccharolytic bacteria, such as *Bifidobacteria* and *Lactobacilli* [51].

Moreover, proteins from red meat are associated with the maximal expansion of pathobionts, possibly altering the inflammatory balance in the gut [52]. Additionally, alcohol consumption may lead to significant microbiota changes. In alcoholics, significant increases in *Enterobacteriaceae*, *Prevotellaceae*, and *Streptococcaceae* abundance can be detected via the activation of an inflammatory cascade [53]. However, not all alcohol-containing drinks are the same because red wine consumers show a relatively high abundance of polyphenol-induced species [53]. Diet may play a pivotal role in shaping GM composition, thereby increasing the risk of DD occurrence. The complex interplay between diet and GM also significantly impacts IBD pathogenesis [54]. However, most of the data related to the association of specific food components, such as dietary emulsifiers, artificial sweeteners, total fiber, or zinc intake with IBD, are inconsistent or inconclusive, probably because the host’s genetic susceptibility could augment or reduce the effect of specific dietary factors [55,56,57]. Therefore, we believe that the different genetic makeup of patients with DD or IBD is crucial in affecting diet–microbe interactions and, in turn, in determining different patterns of dysbiosis and subsequent gut inflammation.

### 5.4. Comorbidities and Polypharmacy

It is well known that the risk of DD complications increases in multimorbid patients. Moreover, multimorbidity, when two or more chronic diseases occur, has been recently associated with fecal microbiota dysbiosis [58,59]. Multimorbidity, in turn, is frequently associated with polypharmacy: chronically taking five or more drugs. Polypharmacy is linked with intestinal dysbiosis, mainly when drugs, such as opioids and neuroleptics, influence colonic motility [59,60]. However, further investigation is needed to discover the exact impact of these drugs on the specific changes in GM. In this regard, we must also consider the possible effects of therapies for IBD on the composition of the GM. Both the anti-TNF-α monoclonal antibodies infliximab and adalimumab and other biological therapies used in treating patients with IBD, such as ustekinumab and vedolizumab, can reduce intestinal dysbiosis by modifying the composition and function of the microbiota [61,62,63,64].

## 6. How Microbiota Changes Impact DD Treatment: The Role of Probiotics

This review aims to address the possible effect of probiotics in treating or preventing intestinal dysbiosis in DD. At the same time, we refer readers to other specific publications on the therapeutic role of antibiotics or intestinal disinfectants, such as rifaximin, in DD. According to the World Health Organization, probiotics are defined as “live microorganisms which, if administered in the right amount, benefit the host.” Specific requirements define a probiotic: a probiotic must have the ability to survive in the gastrointestinal tract, adhere to the mucosa epithelium, be resistant to gastric acids and bile, and must be free of transferable genes of antibiotic resistance [65]. The rationale for using probiotics in the treatment of DD is due to various factors, such as the ability to produce antimicrobials (for example, clausin and reuterin), the competitive metabolic interactions with pro-inflammatory organisms, the inhibition of adherence and translocation for different pathogens, and the pro-kinetic properties reported for some strains.

Moreover, they may also influence the mucosal immunity defense at the epithelial level, decreasing the activity of several pro-inflammatory cytokines [66]. In addition, some specific strains can also maintain adequate bacterial colonization of the gastrointestinal tract, inhibiting colonic bacterial overgrowth and pathogen metabolism. In this way, these bacterial strains may increase both the anti-inflammatory effects and the capability to enhance anti-infection defenses [67].

Several probiotic strains have been tested in the management of different phases of DD. These studies have tested single strains, such as Lactobacillus *paracasei B21060* and *F19* and *Escherichia coli Nissle 1917*, or probiotic mixtures, such as the so-called De Simone Formulation (DSF) containing *S. thermophilus*, *B. longum*, *B. breve*, *B. infantis*, *L. acidophilus*, *L. plantarum*, *L. paracasei*, and *L. delbrueckii* subsp. *Bulgaricus*. These studies have shown significant efficacy in controlling SUDD symptoms and preventing AD recurrence [68]. However, recent double-blind, placebo-controlled studies are now opening new horizons regarding the use of probiotics in DD.

### 6.1. Probiotics in SUDD

Recently, Kvasnovsky et al. conducted a double-blind, placebo-controlled trial on 143 patients suffering from SUDD [69]. The active arm was treated with a probiotic mixture containing *L. rhamnosus*, *E. faecium*, *L. acidophilus*, and *L. plantarum* (1 mL/kg/day). The primary endpoint of this study was the reduction of abdominal pain. After three months of treatment, the authors found a significant decrease in some symptoms, such as constipation, diarrhea, mucorrhea, and back pain, in the group supplemented with probiotics, while abdominal pain was decreased in both groups without any significant difference [69]. Another older multicenter double-blinded placebo-controlled trial assessed the effectiveness of mesalazine (1.6 g/die) and probiotics (*L. casei DG* 24 billion/die) versus placebo in maintaining remission from SUDD [70]. The three treatments were administered for 10 days each month over 12 months. This bacterial strain was chosen because it had previously been shown to colonize the human intestine, resist hydrochloric acid and bile salts, persist in the gastrointestinal tract for approximately two weeks after discontinuation of treatment, and be effective against gram-negative anaerobes [71]. *L. casei* was significantly better than placebo in maintaining SUDD remission, especially in combination with mesalazine. Moreover, both mesalazine and *L. casei*, alone or in combination, were significantly better than the placebo in preventing the occurrence of acute diverticulitis [70].

### 6.2. Probiotics in AUD

A first double-blind, placebo-controlled trial was conducted in 88 patients with a diagnosis of AUD [72]: group A (44 patients) was treated with ciprofloxacin 400 mg/bid and metronidazole 500 mg/tid for one week, plus *L. reuteri ATCC PTA 4659*/bid for 10 days. Group B (44 patients) was treated with the same antibiotic therapy for one week, plus placebo/bid for 10 days. The authors found that *L. reuteri strain 4659*, when administered after antibiotics, significantly reduced abdominal pain and inflammatory markers compared with the placebo group, even within three days of administration (pain decreasing vs. placebo: *p* < 0.0001; C-reactive protein (CRP) value reducing vs. placebo: *p* < 0.0001) [72]. Moreover, the patients treated with probiotic supplementation had a shorter hospital stay vs. placebo supplementation (*p* < 0.0001) [72]. The same group recently conducted a more interesting double-blind, randomized, placebo-controlled trial in 119 patients with AUD [73]. The probiotic group (61 patients) was treated with fluids, bowel rest, and *L. reuteri*/bid for ten days, and the placebo group (58 patients) was treated with the same therapy and placebo/bid for 10 days. All patients completed a visual analog scale (VAS) daily for abdominal pain. After three days, both groups had similar VAS score reduction for abdominal pain. However, the CRP value and the fecal calprotectin levels were significantly decreased in the probiotic group vs. the placebo group (*p* < 0.05). Finally, the placebo group had a more extended hospital stay than the probiotic group (83.5 h vs. 75.5 h, *p* < 0.05) [73].

## 7. Conclusions

DD, including SUDD and AD, have dysbiotic landmarks that differ from those typically found in IBD, such as a reduced abundance of *Roseburia* and an increased abundance of *Akkermansia*. Moreover, small, randomized, placebo-controlled studies have demonstrated the efficacy of probiotics in managing the different clinical manifestations of DD. However, current studies on the GM composition in DD still suffer from several biases (in particular, different sampling modalities and various microbiota searching techniques). Further studies are needed to confirm whether the currently detected imbalance in the GM is a cause or effect of the different clinical expressions of DD.

## Figures and Tables

**Table 1:** Microbiota characterization in diverticulosis.

Authors (Years) [Reference]	Study Design	Overall Study Population	Sampling Methods	Detection Methods	Microbiome Association with Diverticulosis	Treatment	Main Findings
Jones (2018)	Case-control	535 consecutive patients (226 with diverticulosis and 309 controls).	Mucosal sampling during colonoscopy.	PCR metagenomics	Proteobacteria and Comamondaceae	None	No significant differences between cases and controls except for a slight reduction in Proteobacteria and Comamondaceae species among cases.
van Rossen (2021)	Case-control	43 consecutive patients (19 with diverticulosis and 24 controls).	Mucosal sampling during colonoscopy.	16S rRNA	None	None	No significant differences between cases and controls.

**Table 2:** Microbiota characterization in symptomatic uncomplicated diverticular disease (SUDD).

Authors (Years) [Reference]	Study Design	Overall Study Population	Sampling Methods	Detection Methods	Microbiome Association with Diverticular Disease	Treatment	Main Findings
Ponziani (2016)	Case-control	20 consecutive patients (4 SUDD, 4 IBS, 4 CD, 4 UC, 4 HE)	Stool	16S rRNA	Roseburia and Colinsella	Rifaximin	Significant increase in Roseburia and Colinsella (which decreased after rifaximin treatment) in SUDD). Significant reduction in Lactobacilli (which increased after rifaximin treatment) in SUDD.
Tursi (2016)	Case-control	44 consecutive patients (only women: 15 SUDD, 13 diverticulosis, 16 controls)	Stool	Real-time PCR	A. muciniphila	None	Significant increase in Akkermansia in SUDD compared with controls.
Barbara (2017)	Case-control	38 consecutive patients (8 SUDD, 14 diverticulosis, 16 controls)	Mucosal sampling during colonoscopy	16S rRNA	Clostridium Cluster IX, Fusobacterium, and Lactobacillaceae	None	Significant decrease in Clostridium Cluster IX, Fusobacterium, and Lactobacillaceae in SUDD; A. muciniphila reduced in the diverticular district compared with the colonic district without diverticula.
Lopetuso (2018)	Case-Control	28 consecutive patients (4 SUDD, 3 IBS, 10 CD, 8 UC, 8 controls)	Stool	16S rRNA	Bacteroides fragilis, Colinsella, and Riminococcaceae	None	Ruminococcaceae increased in SUDD, with depletion of Colinsella and Bacterioidetes fragilis.
Linninge (2018)	Case-control	51 consecutive patients (16 SUDD and 24 controls)	Mucosal sampling during colonoscopy	PCR-based profiling	Enterobacteriaceae	None	Increased abundance of Enterobacteriaceae in SUDD compared with controls.
Kvasnovsky (2018)	Cohort	30 consecutive SUDD patients (15 PD-SUDD and 15 NPD-SUDD)	Stool	16S rRNA	Pseudobutyrivibrio Bifidobacterium, Christensenellaceae, Mollicutes RF9, Bacteroides, Faecalibacterium, and Ruminococcus	None	Bacteroides, Faecalibacterium, and Ruminococcus increased in all SUDD patients. - Cyanobacterium was associated with pain score. - Pseudobutyrivibrio Bifidobacterium, Christensenellaceae, and Mollicutes RF9 increased in PD-SUDD vs. NPD-SUDD.
Laghi (2018)	Cohort	13 consecutive SUDD patients (15 PD-SUDD and 15 NPD-SUDD)	Stool	Real-time PCR	Akkermansia muciniphila	Probiotic mixture, rifaximin, mesalazine, fibers	Significant decrease in Akkermansia under treatment, parallel with symptom improvement. - Increase in Akkermansia during pharmacological washout, parallel with symptom recurrence.
Ponziani (2020)	Case-control	25 consecutive patients (7 SUDD, 8 IBS, 5 CD, 5 UC)	Stool	16S rRNA	Faecalibacterium, Ruminococcus, and Roseburia	Rifaximin	Significant increase in Fecalibacterium and a significant decrease in Roseburia and Ruminococcus in patients responding to rifaximin.
Tursi (2022)	Case-control	44 consecutive patients (only women: 15 SUDD, 13 diverticulosis, 16 controls)	Stool	Real-time PCR	F. prausnitzii	None	Slight increase (not significant) in *F. prausnitzii* in SUDD compared with controls.

Abbreviations: SUDD, symptomatic uncomplicated diverticular disease; PD-SUDD, post diverticulitis symptomatic uncomplicated diverticular disease; NPD-SUDD, non-post diverticulitis symptomatic uncomplicated diverticular disease; HE, hepatic encephalopathy; IBS, irritable bowel syndrome; CD, Crohn’s disease; UC, ulcerative colitis.

**Table 3:** Microbiota characterization in acute diverticulitis (AD).

Authors (Years) [Reference]	Study Design	Overall Study Population	Sampling Methods	Detection Methods	Microbiome Association with Acute Diverticulitis	Treatment	Main Findings
Gueimonde (2007)	Cohort	34 patients (21 CRC, 9 AD, 4 IBD)	Mucosal biopsy during surgery	16S rRNA microbial profiling	Bifidobacterium longum	Surgery	*B. longum* significantly increased in AD vs. CRC and IBD.
Daniels (2014)	Case-control	56 patients (31 AUD and 25 controls)	Rectal swab	PCR based	Enterobacteriaceae	None	*Enterobacteriaceae* abundant in AD.
Schieffer (2017)	Cohort	Nine patients (resected diverticulitis)	Mucosal biopsy on surgical specimen	16S rRNA	Microbacteriaceae	Surgery	The mucosal microbiota associated with disease overexpressed *Microbacteriaceae* compared with the microbiota related to adjacent tissue.
O’Grady (2022)	Case-control	82 patients (44 AUD, 21 ACD, 27 controls)	Rectal swab	16S rRNA	*Lachnospiraceae*, *Ruminococcus*, and *Faecalibacterium Fusobacteria*, *Prevotella*, and *Paraprevotella*	None	*Lachnospiraceae*, *Ruminococcus*, and *Faecalibacterium* decreased in AD; *Fusobacteria*, *Prevotella*, and *Paraprevotella* increased in AD; *Prevotella*, *Fusicatenibacter*, and *Faecalibacterium* more abundant in ACD than in AUD.

Abbreviations: CRC, colorectal cancer; AD, acute diverticulitis; AUD, acute uncomplicated diverticulitis; ACD, acute complicated diverticulitis; IBD, inflammatory bowel disease.

## Data Availability

Not applicable.
